# Relationship between cumulative exposure to triglyceride-glucose index and heart failure: a prospective cohort study

**DOI:** 10.1186/s12933-023-01967-5

**Published:** 2023-09-04

**Authors:** Huancong Zheng, Guanzhi Chen, Kuangyi Wu, Weiqiang Wu, Zegui Huang, Xianxuan Wang, Zekai Chen, Zefeng Cai, Zhiwei Cai, Yulong Lan, Shouling Wu, Youren Chen

**Affiliations:** 1 Department of Cardiology, Second Affiliated Hospital of Shantou University Medical College, 69 Dongxia North Road, Shantou, 515000 China; 2 Shantou University Medical College, Shantou, China; 3 Cardiac Arrhythmia Center, Fuwai Hospital, National Center for Cardiovascular Diseases, Chinese Academy of Medical Sciences and Peking Union Medical College, Beijing, China; 4 Department of Cardiology, Sun Yat-sen Memorial Hospital of Sun Yat-sen University, Guangzhou, China; 5 Department of Epidemiology, University Medical Center Groningen, University of Groningen, Groningen, the Netherlands; 6 Centre for Precision Health, Edith Cowan University School of Medical and Health Sciences, Joondalup, Australia; 7 Department of Cardiology, Kailuan General Hospital, 57 Xinhua East Road, Tangshan, 063000 China

**Keywords:** Triglyceride-glucose index, Cumulative exposure, Insulin resistance, Heart failure

## Abstract

**Background:**

High triglyceride-glucose index (TyG) is a major risk factor for heart failure, but the long-term effect of high TyG index on the risk of developing heart failure remains unclear. Therefore, we aimed to determine the relationship between the cumulative exposure to TyG index and the risk of heart failure.

**Methods:**

A total of 56,149 participants from the Kailuan Study, who participated in three consecutive health examinations in 2006, 2008, and 2010 and had no history of heart failure or cancer were recruited for this study. The cumulative TyG index was calculated as the weighted sum (value × time) of the mean TyG index for each time interval. The participants were placed into quartiles based on their cumulative TyG index. The study ended on December 31, 2020, and the primary outcome was new-onset heart failure during the follow-up period. In addition, a Cox proportional hazards regression model and a restricted cubic spline analysis were used to further evaluate the relationship between cumulative TyG index and the risk of heart failure.

**Results:**

During a median follow-up period of 10.04 years, a total of 1,312 new heart failure events occurred. After adjustment for potential confounding factors, the Cox regression analysis showed that the hazard ratios (95% confidence intervals) for the risk of heart failure in the Q2, Q3, and Q4 groups were 1.02 (0.83,1.25), 1.29 (1.07,1.56) and 1.40 (1.15,1.71), respectively, vs. the Q1 group. The subgroup analysis showed a significant interaction between cumulative TyG index and BMI or waist circumference, but there was no interaction between age, sex and cumulative TyG index. The restricted cubic spline analysis showed a dose-response relationship between cumulative TyG index and the risk of heart failure. In addition, the sensitivity analysis generated results that were consistent with the primary results.

**Conclusions:**

High cumulative TyG index is associated with a higher risk of heart failure. Thus, the TyG index may be useful for the identification of individuals at high risk of heart failure. The present findings emphasize the importance of the long-term monitoring of the TyG index in clinical practice.

**Supplementary Information:**

The online version contains supplementary material available at 10.1186/s12933-023-01967-5.

## Background

Heart failure (HF) is a common disease worldwide, with high incidence and mortality rates [1]. The prevalence of heart failure in developed countries is estimated to be 1–2%, and the number of people with HF worldwide increased from 33.5 million in 1990 to 64.3 million in 2017 [2, 3]. The American Heart Association has stated that as the prevalence of HF continues to rise, the associated economic and social burdens increase. In China, the prevalence rate of HF rose from 0.9% to 2000 to 1.3% in 2015 [4], and this rise has placed an enormous burden on the national healthcare system. Therefore, the risk factors for HF should be better understood to implement effective preventive measures [5].

Insulin resistance (IR) is an important mechanism for the development of type 2 diabetes [6] and has been shown in numerous studies to be an independent risk factor for cardiovascular disease (CVD) [7, 8]. The hyperinsulinemic-euglycemic clamp technique is considered to be the gold-standard method for the evaluation of insulin resistance [9], but its implementation requires complex equipment and skilled personnel; and it is time-consuming, expensive, requires multiple blood samples, and is not readily accepted by patients. Therefore, it cannot be used on a large scale in clinical practice [10]. In contrast, the triglyceride-glucose index (TyG), a reliable alternative index of IR, can be calculated using only the patient’s fasting blood glucose (FBG) and fasting triglyceride (TG) concentrations [11, 12]. A number of recent studies have shown that high TyG index is positively associated with the risk of CVD, which includes myocardial infarction, stroke, and atherosclerosis [13–15], while others have shown that high TyG index is a risk factor for HF [16]. However, most previous studies of the relationship between TyG index and HF have used single measurements, and therefore information on the relationship between the long-term change in TyG index and the risk of HF is limited. Consequently, in the present study, we used a large community-based prospective cohort from the Kailuan study to evaluate the relationship of cumulative TyG index with HF.

## Methods

### Study population

**Fig. 1:**
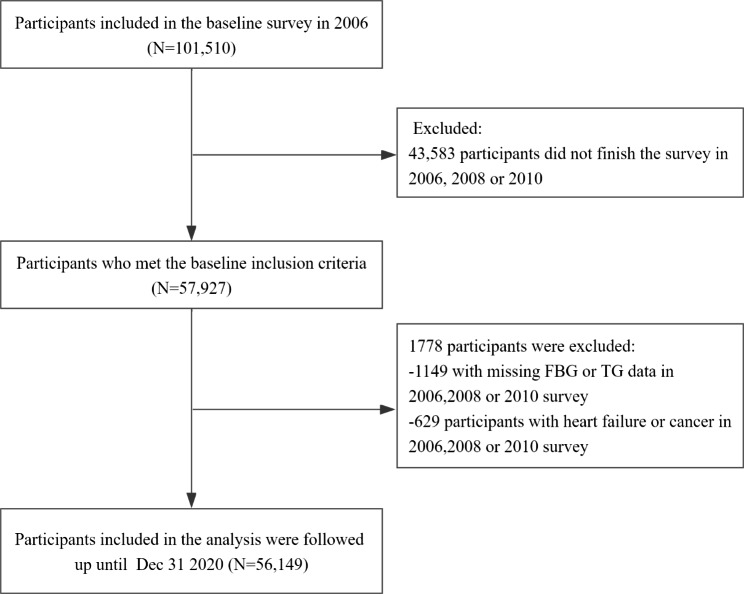
Flow chart of inclusion and exclusion in this study

The Kailuan study is a large community-based prospective cohort study that is being conducted in Tangshan, China and aims to identify the risk factors for chronic non-communicable diseases. The research design and methods of the Kailuan study have been described previously [17, 18]. In brief, the Kailuan study started in 2006–2007, a total of 101,510 participants were recruited for the baseline survey, and they were followed up every 2 years. Comprehensive surveys were conducted on each occasion that comprised the completion of a questionnaire, a physical examination, and biochemical evaluation, including of FBG, and serum TG, high-density lipoprotein-cholesterol (HDL-C), and low-density lipoprotein-cholesterol (LDL-C). To date, eight rounds of health assessments have been completed. Participants who met the following conditions were included in the present study: (1) the completion of three health check-ups between 2006 and 2010; (2) complete data for blood glucose and TG from at least three check-ups; and (3) the provision of written informed consent. Participants with a history of HF or tumor prior to the third check-up (2010–2011) were excluded. Data from a total of 56,149 participants were ultimately included (Fig. 1). The study was conducted in accordance with the principles of the Declaration of Helsinki and approved by the Ethics Committee of Kailuan General Hospital (200,605).

### Data collection and definitions

The participants completed the survey at 11 hospitals in Kailuan community. Trained staff used structured questionnaires to collect the demographic information (such as their age, sex, and education), medical history (such as of hypertension, diabetes, arrhythmia and myocardial infarction), and lifestyle factors (such as smoking status, alcohol consumption, and physical activity) of the participants, and their anthropometric measurements were reported previously [19].

Hypertension was defined as a systolic blood pressure (SBP) of ≥ 140 mmHg and/or diastolic blood pressure (DBP) of ≥ 90 mmHg, and/or a clear history of hypertension and/or the use of antihypertensive medication [20]. Diabetes was defined as a fasting plasma glucose (FPG) concentration of ≥ 7.0 mmol/L, and/or a clear history of diabetes, and/or the use of hypoglycemic drugs [21]. A normal waist circumference (WC) was defined as < 90 cm for men and < 80 cm for women [22]. Smoking and drinking habits were recorded as present or absent; physical activity was defined as exercising at least 4 times/week, with each session lasting at least 20 min; and body mass index (BMI) was calculated as body mass in kilograms divided by height in meters, squared.

### Biochemical measurements and TyG index

Blood samples were collected after fasting for 8–12 h. On the day of the physical examination, 5 ml of venous blood was drawn from an elbow by a laboratory technician between 07:00 and 09:00 and analyzed using a Hitachi 747 automatic analyzer (Hitachi, Tokyo, Japan). The serum TC, HDL-C, LDL-C, FBG, TG, and high-sensitivity C-reactive protein (hs-CRP) concentrations were measured. Creatinine levels were measured by a creatine oxidase assay (Creatine Kit, BioSino BioTechnology and Science Inc, Beijing, China) [23]. Estimated glomerular filtration rate (eGFR) was calculated using the modified four-variable Chronic Kidney Disease Epidemiology Collaboration formula, with an adjustment factor of 1.1 for the Chinese population [24].

The TyG index was calculated as ln [TG (mg/dL) × FBG (mg/dL)/2] [25], and the cumulative TyG index (cumTyG) was calculated as the weighted sum of the mean TyG index values obtained at each visit as: (TyG index_2006_ + TyG index_2008_)/2 × time 1–2 + (TyG index_2008_ + TyG index_2010_)/2 × time 2–3, where the TyG index in 2006, TyG index in 2008, and TyG index in 2010 are the TyG indices calculated at the first, second, and third visits, respectively, and times 1–2 and 2–3 are the specific time intervals in years between consecutive examinations [26].

### Assessment of heart failure

The health examination in 2010 was regarded as the start of the follow-up period and the first identification of an HF event was regarded as the endpoint of follow-up. An HF assessment was performed annually during the follow-up period. Potential HF was identified using hospital discharge summaries, death certificates, and social medical insurance records. The diagnostic criteria for HF were the Chronic Heart Failure Diagnosis Guidelines of the European Society of Cardiology [27]. The following conditions were identified by reviewing the participants’ medical records: (1) clinical symptoms of heart failure, including dyspnea, fatigue, fluid retention, New York Heart Association (NYHA) functional classification II, III, or IV, and Killip functional classification II, III, and IV; (2) left ventricular ejection fraction, measured on two-dimensional and Doppler echocardiography using the modified Simpson’s method, of ≤ 50%; and (3) high plasma natriuretic peptide (NT-proBNP) concentration. A diagnosis of HF was made if condition (1) and either condition (2) or condition (3) were fulfilled. For participants who experienced two or more episodes of HF, the timing and nature of the first episode were used as the outcomes; and for those who did not experience any HF events during the follow-up period, December 31, 2020 or the date of death was recorded as the endpoint, as appropriate.

### Statistical analysis

Normally distributed continuous data are described as mean ± standard deviation, non-normally distributed continuous data are described as median and interquartile range, and categorical data are described using frequency and percentage. To compare the characteristics of the cumulative TyG index groups, the chi-square test was used for categorical data, and one-way ANOVA or the Kruskal–Wallis test was used for continuous data. The cumulative incidence was calculated using Kaplan–Meier method, and groups were compared using the log-rank test. Missing data for covariates were imputed using the multiple imputation method. The incidence density of the event is expressed as the number of events per 1,000 person-years, and was calculated by dividing the number of events by the total number of person-years of follow-up.

To evaluate the independent risk correlation between the cumulative TyG index and HF, multivariate Cox proportional hazards regression analysis was performed, with adjustment for age, sex, heart rate, WC, BMI, LDL-C, HDL-C, hs-CRP, eGFR, current smoking status (yes or no), current alcohol consumption (yes or no), physical activity status (yes or no), the presence of hypertension (yes or no), the presence of diabetes (yes or no), history of myocardial infarction (yes or no), history of arrhythmia (yes or no), the use of anti-hypertensive drugs (yes or no), the use of hypoglycemic drugs (yes or no), and the use of lipid-lowering drugs (yes or no). Subgroup analyses was performed after stratification of the participants by age (< 60 years vs. ≥60 years), sex (male vs. female), BMI (normal weight: <25 kg/m^2^vs. overweight or obesity: ≥25 kg/m^2^) [28] and WC (men < 90 cm or women < 80 cm vs. men ≥ 90 cm or women ≥ 80 cm). In addition, to evaluate the long-term risk of exposure to TyG index with respect to HF, we used the American College of Cardiology (ACC)/American Heart Association (AHA) model for the prediction of HF risk [29].

Finally, several sensitivity analyses were performed to test the robustness of the results. First, to account for the risk of reverse causality, participants who had experienced an HF event within the first year were excluded. Second, to account for the potential effect of the post-infarction condition on HF, participants with a history of myocardial infarction were excluded. Third, the incident HF analysis was repeated with the Fine-Gray model, considering death from non-HF as a competing risk [30]. Finally, to account for the effects of drug use on HF outcomes, three sensitivity analyses were performed separately with respect to the use of anti-hypertensive drugs, hypoglycemic agents, and lipid-lowering drugs. Statistical analyses were performed using SAS v.9.4 (SAS Institute, Inc, Cary, NC, USA). A two-sided *P*-value < 0.05 was considered to represent statistical significance.

## Results

### Baseline characteristics of the participants

A total of 57,927 participants attended all three of the medical examinations in 2006, 2008, and 2010. Of these, 1,149 individuals with missing FBG and TG data were excluded, as were 629 with a history of HF or cancer at the 2006 medical examination or who developed one of these between 2006 and 2010. Therefore, data from 56,149 participants were analyzed. These participants comprised 43,013 (76.6%) men and 13,136 (23.4%) women, with a mean age of 49.42 ± 11.93 years. The baseline characteristics of the participants, categorized according to the cumTyG quartile, are shown in Table 1. Compared with the lowest quartile group, participants with higher cumTyG indices were more likely to be male; were older; had lower eGFR; had higher BMI, SBP and DBP; had higher FBG, TG, LDL, and hs-CRP concentrations; and were more likely to have diabetes, hypertension, history of myocardial infarction and history of arrhythmia.

**Table 1:** Baseline characteristics of participants by cumulative TyG index quartile

	Total	Quartiles of cumulative TyG index				P
		Q1(22.11–32.06)	Q2(32.06–34.53)	Q3(34.53–37.60)	Q4(37.60-56.14)	
N	56,149	14,037	14,038	14,037	14,037	-
Age, years	49.42 ± 11.93	45.07 ± 10.64	47.37 ± 11.62	51.15 ± 11.93	54.09 ± 11.43	< 0.01
Male, N (%)	43,013(76.6)	10,016(71.4)	11,219(79.9)	10,969(78.1)	10,809(77.0)	< 0.01
SBP, mmHg	129.88 ± 17.06	124.01 ± 15.82	128.67 ± 15.86	132.14 ± 17.04	134.69 ± 17.53	< 0.01
DBP, mmHg	83.68 ± 9.25	81.35 ± 9.21	83.59 ± 8.91	84.58 ± 9.21	85.21 ± 9.21	< 0.01
BMI, kg/m^2^	25.04 ± 3.16	24.07 ± 3.06	24.87 ± 3.13	25.32 ± 3.10	25.91 ± 3.07	< 0.01
WC, cm	87.35 ± 8.58	84.46 ± 8.55	86.89 ± 8.34	88.32 ± 8.35	89.72 ± 8.18	< 0.01
HDL-C, mmol/L	1.54 ± 0.32	1.59 ± 0.32	1.55 ± 0.30	1.53 ± 0.32	1.48 ± 0.33	< 0.01
LDL-C, mmol/L	2.50 ± 0.64	2.33 ± 0.64	2.49 ± 0.60	2.54 ± 0.63	2.63 ± 0.64	< 0.01
eGFR, ml/min/1.73m^2^	87.16 ± 17.67	90.39 ± 16.88	87.28 ± 18.46	85.64 ± 18.38	85.34 ± 16.41	< 0.01
TG, mmol/L	1.68 ± 1.26	1.07 ± 0.51	1.46 ± 0.71	1.79 ± 1.11	2.41 ± 1.84	< 0.01
FBG, mmol/L	5.56 ± 1.40	5.10 ± 0.68	5.36 ± 0.97	5.59 ± 1.33	6.18 ± 2.01	< 0.01
hs-CRP, mg/L	2.63 ± 3.70	2.61 ± 3.75	2.54 ± 3.90	2.66 ± 3.84	2.70 ± 3.28	< 0.01
TyG index_2006_	8.65 ± 0.69	8.22 ± 0.53	8.57 ± 0.58	8.74 ± 0.65	9.05 ± 0.71	< 0.01
TyG index_2008_	8.67 ± 0.69	8.20 ± 0.51	8.57 ± 0.51	8.77 ± 0.61	9.16 ± 0.71	< 0.01
TyG index_2010_	8.70 ± 0.68	8.31 ± 0.51	8.61 ± 0.56	8.78 ± 0.65	9.10 ± 0.71	< 0.01
CumTyG	35.02 ± 4.22	30.09 ± 1.63	33.29 ± 0.71	35.98 ± 0.88	40.70 ± 2.62	< 0.01
Current smoker, N (%)	21,496(38.3)	5556(39.6)	5651(40.3)	5311(37.8)	4978(35.5)	< 0.01
Current drinker, N (%)	19,735(35.1)	5005(35.7)	5090(36.3)	4890(34.8)	4750(33.8)	< 0.01
Physical activity, N (%)	8221(14.6)	1655(11.8)	1710(12.2)	2280(16.2)	2576(18.4)	< 0.01
History of myocardial infarction, N (%)	506(0.90)	87(0.62)	83(0.59)	118(0.84)	218(1.56)	< 0.01
History of arrhythmia, N (%)	2081(3.71)	469(3.34)	446(3.18)	538(3.83)	628(4.47)	< 0.01
Hypertension, N (%)	27,429(48.9)	4947(35.2)	6362(45.3)	7569(53.9)	8551(60.9)	< 0.01
Diabetes mellitus, N (%)	6511(11.6)	499(3.55)	996(7.10)	1733(12.3)	3283(23.4)	< 0.01
Hypoglycemic drugs, N (%)	9110(16.2)	1479(10.5)	2144(15.3)	2575(18.3)	2912(20.7)	< 0.01
Anti-hypertensive drugs, N (%)	3466(6.17)	255(1.82)	498(3.55)	860(6.13)	1853(13.2)	< 0.01
Lipid-lowering drugs, N (%)	1692(3.01)	248(1.77)	306(2.18)	417(2.97)	721(5.14)	< 0.01

SBP systolic blood pressure, DBP diastolic blood pressure, BMI body mass index, WC waist circumference, HDL-C high-density lipoprotein cholesterol, LDL-C low-density lipoprotein cholesterol, TG triglyceride, FBG fasting blood glucose, hs-CRP high-sensitivity C reactive protein, eGFR estimated glomerular filtration rate, TyG index triglyceride-glucose index

### Relationship between cumulative TyG index and the risk of heart failure

**Fig. 2:**
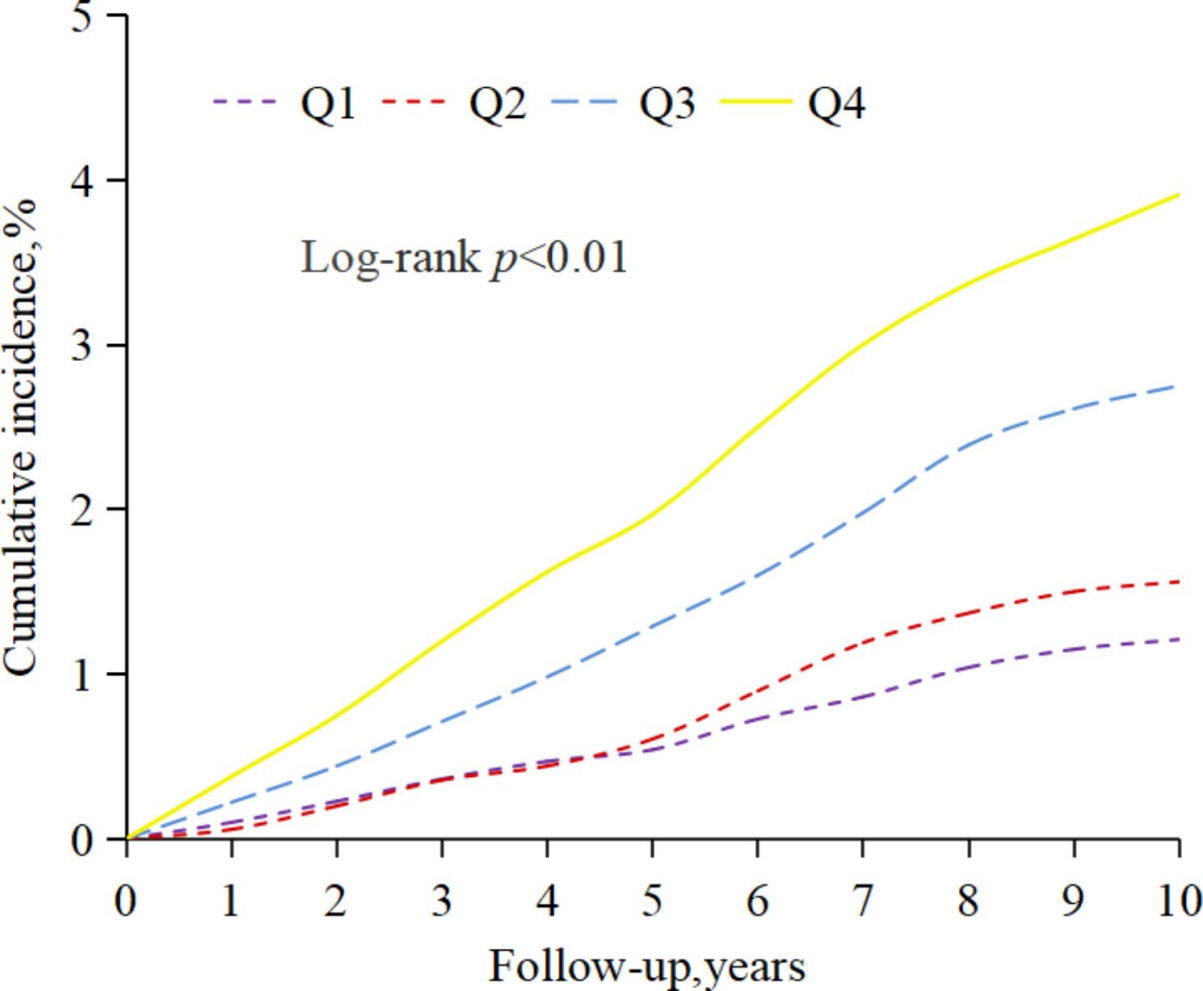
Kaplan–Meier incidence rate of heart failure by cumTyG index

**Fig. 3:**
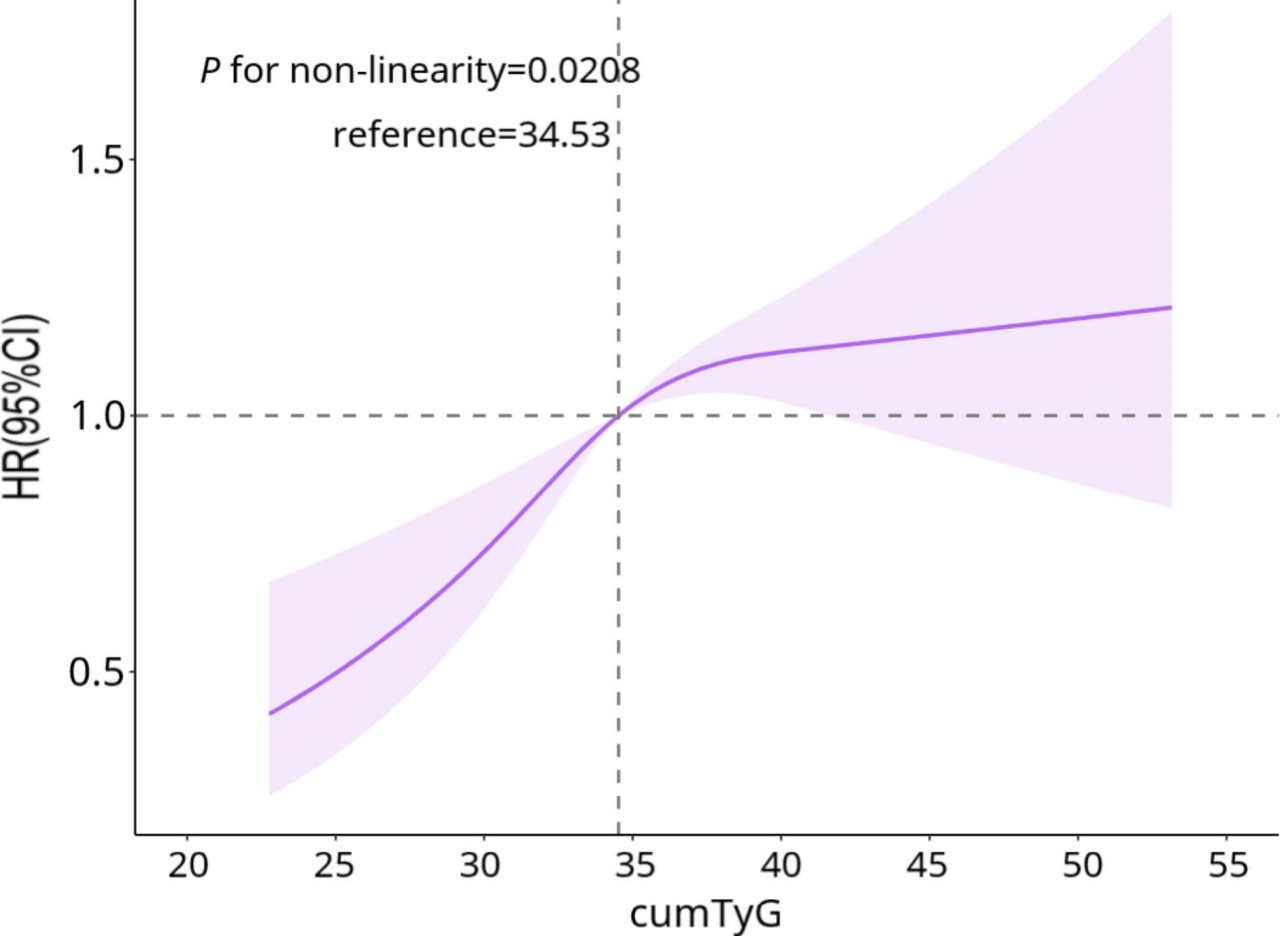
The associations of cumTyG index with risk of heart failure Caption: Cox regression models with restricted cubic splines were fitted to the data with 3 knots at the 25th, 50th and 75th percentiles of the cumTyG index. TyG index, triglycerides-glucose index, HR hazard ratio, CI confidence interval. The solid line represents the point estimate of cumTyG index correlation with the risk of heart failure, and the shaded part represents the 95% CI estimate. Covariates in the model include age, sex, heart rate, HDL-C, LDL-C, WC, hs-CRP, eGFR, current smoker, current drinker, physical activity, hypertension, diabetes mellitus, history of myocardial infarction, history of arrhythmia, hypoglycemic drugs, anti-hypertensive drugs, lipid-lowering drugs and the TyG index_2010_ at baseline

During a median follow-up of 10.04 years (interquartile range, 9.70–10.32), 1,312 participants experienced HF events. According to the cumulative TyG quartiles, the incidences of HF in the Q1, Q2, Q3, and Q4 groups were 1.19, 1.57, 2.80, and 4.03 per 1,000 person-years, respectively (Table 2). The Kaplan–Meier curve showed that the incidence of HF gradually increased from the Q1 to the Q4 group. The cumulative incidence of HF in each group was significantly different among the groups (*P* < 0.01, Fig. 2), according to the log-rank test. After adjustment for potential confounding factors, the fully adjusted hazard ratios (HRs) and 95% confidence intervals (CIs) in Model 4 for the Q2–Q4 groups vs. the Q1 group were 1.02 (0.83,1.25), 1.29 (1.07,1.56) and 1.40 (1.15,1.71), respectively. In addition, restricted cubic spline analysis showed that there was a dose-response relationship between the cumulative TyG index and the risk of HF (*P* for non-linearity = 0.0208) (Fig. 3).

**Table 2:** Association of cumulative TyG index with heart failure

	Quartiles of cumulative TyG index				P for trend
	Q1(22.11–32.06)	Q2(32.06–34.53)	Q3(34.53–37.60)	Q4(37.60-56.14)	
Case/Total	170/14,037	222/14,038	385/14,037	535/14,037	
Incidence rate, per 1000 person-years	1.19	1.57	2.80	4.03	
Model 1	1(Reference)	1.12(0.92,1.37)	1.59(1.32,1.91)	1.94(1.62,2.32)	< 0.001
Model 2	1(Reference)	1.03(0.84,1.25)	1.31(1.08,1.58)	1.43(1.18,1.72)	< 0.001
Model 3	1(Reference)	1.02(0.84,1.25)	1.30(1.08,1.57)	1.41(1.17,1.71)	< 0.001
Model 4	1(Reference)	1.02(0.83,1.25)	1.29(1.07,1.56)	1.40(1.15,1.71)	< 0.001
Model 5	1(Reference)	1.01(0.82,1.23)	1.27(1.05,1.53)	1.36(1.11,1.65)	< 0.001

Model 1: adjust for age, sex

Model 2: included variables in model 1 and further heart rate, HDL-C, LDL-C, WC, hs-CRP, eGFR, current smoker, current drinker, physical activity, hypertension, diabetes mellitus, history of myocardial infarction and history of arrhythmia

Model 3: included variables in model 2 and further hypoglycemic drugs, anti-hypertensive drugs and lipid-lowering drugs

Model 4: included variables in model 3 and further the TyG index_2010_ at baseline

Model 5: included variables in model 3 and further the TyG index_2006_ at baseline

### Relationship between baseline TyG index and the risk of heart failure

The incident rate presented an increas-ing trend in four groups divided by the quartiles of baseline TyG index, which were 1.57, 2.00, 2.69, and 3.21 per 1000 person-years, respectively (Additional file 1, Table S1). The Kaplan–Meier curve showed that the incidence of HF gradually increased from the Q1 to the Q4 group. The cumulative incidence of HF in each group was significantly different among the groups (*P* < 0.01) (Additional File 2, Fig. S1), according to the log-rank test. After adjustment for potential confounding factors, the fully adjusted hazard ratios (HRs) and 95% confidence intervals (CIs) in Model 3 for the Q2–Q4 groups vs. the Q1 group were 1.08 (0.91,1.30), 1.23 (1.04,1.46) and 1.24 (1.04,1.47), respectively.

### Results of the subgroup and sensitivity analyses

The relationship between the cumulative TyG index and HF was stratified according to age, sex, BMI, and WC (Table 3), and we found a significant interaction between cumulative TyG index and both BMI and WC, but not between age, sex and cumulative TyG index. The sensitivity analysis, in which we adjusted for covariates and excluded participants who experienced an HF event within 1 year and those with a history of myocardial infarction (Additional file 1, Tables S2 and S3), generated results that were consistent with the main findings. Considering the competing risks of non-heart failure death, we used the Fine-Gray model to assess the association of cumulative TyG index with new-onset heart failure, and the results remained stable (Additional file 1, Table S4). Furthermore, the results were unaffected by the exclusion of participants who were using anti-hypertensive drugs, hypoglycemic drugs, and lipid-lowering drugs (Additional file 1, Table S5).

**Table 3:** Stratified analysis for the association of cumulative TyG index with heart failure

	Quartiles of cumulative TyG index				P for interation
	Q1(22.11–32.05)	Q2(32.05–34.52)	Q3(34.52–37.58)	Q4(37.58–55.41)	
Age					0.08
Age < 60years					
Case/Total	113/13,001	143/12,283	176/11,081	226/10,014	
	1(Reference)	1.16(0.90,1.49)	1.37(1.07,1.76)	1.59(1.23,2.05)	
Age ≥ 60years					
Case/Total	57/1036	79/1754	209/2957	309/4023	
	1(Reference)	0.75(0.54,1.06)	1.13(0.84,1.52)	1.20(0.88,1.62)	
Sex					0.31
Male					
Case/Total	140/10,016	190/11,219	319/10,969	422/10,809	
	1(Reference)	1.04(0.83,1.29)	1.32(1.07,1.63)	1.45(1.16,1.80)	
Female					
Case/Total	30/4021	32/2818	66/3069	113/3228	
	1(Reference)	0.87(0.52,1.46)	1.21(0.76,1.92)	1.11(0.69,1.80)	
BMI					0.02
BMI < 25 kg/m^2^					
Case/Total	80/8953	96/7602	158/6535	191/5621	
	1(Reference)	1.03(0.76,1.39)	1.47(1.10,1.95)	1.60(1.18,2.17)	
BMI ≥ 25 kg/m^2^					
Case/Total	90/5084	126/6435	227/7503	344/8416	
	1(Reference)	0.96(0.73,1.26)	1.12(0.87,1.45)	1.20(0.92,1.56)	
WC					0.005
Male < 90 or Female < 80 cm					
Case/Total	63/8060	85/6787	125/5708	169/5125	
	1(Reference)	1.17(0.84,1.63)	1.49(1.08,2.06)	1.70(1.22,2.37)	
Male ≥ 90 or Female ≥ 80 cm					
Case/Total	107/5977	137/7250	260/8330	366/8912	
	1(Reference)	0.89(0.69,1.15)	1.14(0.90,1.44)	1.19(0.93,1.53)	

Model adjusted for age, sex, heart rate, HDL-C, LDL-C, WC, hs-CRP, eGFR, current smoker, current drinker, physical activity, hypertension, diabetes mellitus, history of myocardial infarction, history of arrhythmia, hypoglycemic drugs, anti-hypertensive drugs, lipid-lowering drugs and the TyG index_2010_ at baseline

### Incremental predictive value of the change in the cumTyG index

In turn, we added TyG index_2006_, TyG index_2010, and cumTyG to_ the original ACC/AHA prediction model and evaluated its efficacy. Of these, the addition of cumTyG was associated with the highest predictive value for the risk of HF, with a C-index of 76.13%, compared with the original ACC/AHA prediction model (C-index of 75.93%), the 2006 prediction model (C-index of 76.04%), and the 2010 prediction model (C-index of 76.01%), its C-indexes were 0.2%, 0.09%, and 0.12% higher, respectively, and the continuous NRIs were 25.37%, 10.72%, and 17.61% higher, respectively (Additional file 1, Table S6).

## Discussion

The main finding of the present study is that a high cumulative TyG index is a risk factor for new-onset HF. A high cumulative TyG index increases the risk of heart failure development independently of traditional risk factors and exhibits a dose-response relationship. In addition, the risk of HF is BMI- and WC-dependent, such that individuals with a normal BMI and with a normal WC have a higher risk of developing HF. Compared with single TyG index, cumulative TyG index is more likely to be a better predictor of the risk of HF.

We found that cumulative exposure to a high TyG index is an independent risk factor for new-onset HF. In previous studies of the relationship between TyG index and the risk of HF, similar conclusions have been reached. In a prospective study of 138,620 participants, Xu et al. [31]. found that after a median follow-up of 8.78 years, the risk of HF was 24% higher in those with the highest quartile of TyG index than in those with the lowest quartile. Similarly, Li et al. [32]. analyzed 95,996 and 19,345 participants in the Kailuan and Hong Kong cohort studies, and found that a higher TyG index was an independent risk factor for the risk of HF in the general population, with the risk of HF being 22% higher in the highest quartile of TyG index than in the lowest quartile. However, in these studies, TyG was measured once, and therefore the change in TyG over time was not assessed. In the present study, we found that participants with a high cumulative TyG index are at a higher risk of HF and that those in the highest quartile group are at the highest risk, with a multifactor-adjusted HR (95% CI) of 1.40 (1.15,1.71), and therefore cumulative exposure to a high TyG index may be associated with a higher risk of HF than a single exposure. In the present study, we evaluated the effect of a 6-year TyG index assessment on HF, and generated more reliable results.

We also found that the risk of HF is bmi- and waist circumference-dependent. The association between the cumulative TyG index and the risk of HF appears to be stronger in normal BMI, normal waist circumferences. Previous studies have found that individuals with a normal BMI or WC and metabolic abnormalities tend to have a higher risk of cardiovascular disease, which may be the reason why a high TyG index in the normal BMI or WC group leads to a higher risk of heart failure than in the large BMI or WC group [33]. In contrast, in individuals with a large BMI or WC, other risk factors, such as abnormal blood lipid concentrations, may mask the effects of TyG index on the risk of HF [34, 35]. Thus, in summary, exposure to high TyG index is associated with a higher risk of HF in individuals with normal BMI or WC.

This study also found that high baseline tyg index was associated with a higher risk of heart failure, and participants in the highest quartile had the highest risk of heart failure, with a multivariate adjusted HR (95%CI) of 1.24 (1.04,1.47). However, compared with the high cumulative TyG index, baseline high TyG exposure may have a lower risk of heart failure than high cumulative exposure to TyG. In addition, previous cohort studies have shown that the cumulative exposure to risk factors such as TyG index, blood pressure, and adverse lipid profile have more marked effects on outcomes such as cardiovascular disease than a single exposure [36–38], which is consistent with the present findings. We constructed HF risk prediction models that incorporated conventional risk factors and the cumTyG or TyG in 2006 or 2010, and found that the predictive ability of the first was significantly better than those that included single TyG values, which suggests that cumTyG may be a better predictor of the risk of HF.

The mechanisms underpinning the long-term changes in TyG index and its relationship with the risk of HF are not yet clear; IR is likely to be involved. IR increases the risk of many chronic metabolic diseases, such as hypertension, diabetes mellitus, and dyslipidemia, which happen to be risk factors for heart failure [39, 40], and the combined effects of IR and these conditions may further increase the risk of HF. In addition, some previous pathophysiological findings may also explain the relationship between IR and HF. For example, a previous meta-analysis suggested that sodium retention, sympathetic nervous system activation, hyperinsulinemia, IR-related metabolic alterations, and greater sensitivity to angiotensin II may be involved; that higher IR is associated with higher risk of HF; and that there may be a causal relationship between IR and HF, according to the results of a Mendelian randomization study [41–43]. Finally, further evidence of the connection between IR and HF lies in the association between IR and structural abnormalities of the heart, such as left ventricular hypertrophy and greater left ventricular mass, which are associated with a higher risk of developing diastolic HF [44, 45].

The present findings have important clinical implications. IR is a major risk factor for cardiovascular events, including HF, but the gold-standard methods for the assessment of IR are the hyperinsulinemic-euglycemic clamp and the homeostatic model assessment of insulin resistance (HOMA-IR) [46], which are difficult to use in clinical and epidemiological investigations. However, the TyG index, as a reliable alternative marker of IR, is easy to calculate in a clinical setting and is highly sensitive and cheap. Furthermore, the use of electronic medical records has made cumTyG easily accessible, and therefore readily applicable for use as a means of identifying individuals at high risk of HF in the general population.

The principal strength of the present study is that it was a large prospective cohort study with a long follow-up period. The relationship between cumTyG and the risk of HF was investigated by monitoring the long-term changes in TyG index. In addition, we performed subgroup and sensitivity analyses with adjustment for confounding factors, which has rendered the findings more reliable. However, the study also had certain limitations. First, although we adjusted for most potential confounding factors, some residual or unmeasured confounding factors that could have affected the results may remain. In addition, the majority of participants in our study were male coal miners, with female participants being underrepresented. Therefore, these findings may not be directly generalizable to other populations, there is still a need to further validate our results in the future in areas where there is a balanced representation of women and men. Last but not least, because we did not measure IR, such as by calculating HOMA-IR, we could not compare the differential effects of IR and TyG index on the risk of HF. Therefore, further studies are needed to compare the predictive values of the TyG index and HOMA-IR with respect to the risk of HF.

## Conclusions

People with a high cumulative TyG index are at a higher risk of developing HF. This simple index may assist with the early identification of individuals who are at high risk of HF, and the present findings emphasize the importance of the long-term monitoring of TyG index in clinical practice.

### Electronic supplementary material

Below is the link to the electronic supplementary material.

Supplementary Material 1

Supplementary Material 2

## Data Availability

The datasets used and/or analyzed during the present study are available from. the corresponding author on reasonable request.

## Abbreviations

TyG index: Triglyceride-glucose index
IR: Insulin resistance
HF: Heart failure
CVD: Cardiovascular disease
TG: Triglyceride
FBG: Fasting blood glucose
SBP: Systolic blood pressure
DBP: Diastolic blood pressure
BMI: Body mass index
TC: Total cholesterol
HDL-C: High-density lipoprotein-cholesterol
LDL-C: Low-density lipoprotein-cholesterol
hsCRP: High-sensitivity C-reactive protein
eGFR: Estimated glomerular filtration rate
HR: Hazard ratio
CI: Confidence interval. RCS:Restricted cubic spline
