# Supplementary material for: Relationship between cumulative exposure to triglyceride-glucose index and heart failure: a prospective cohort study
Source: Cardiovasc Diabetol. 2023 Sep 4;22:239. doi: 10.1186/s12933-023-01967-5 (PMC10476374; doi:10.1186/s12933-023-01967-5)
Supplement: Supplementary file 1 — Supplementary Material 1 [file 12933_2023_1967_MOESM1_ESM.docx]

**Additional file 1**

**Additional tables**

**Table S1** Association of baseline TyG index with heart failure

**Table S2** Sensitivity analysis of excluding outcome events within the first year of follow-up

**Table S3** Sensitivity analysis of excluding participants with the history of myocardial infarction

**Table S4** Sensitivity analysis of competing risk model

**Table S5** Sensitivity analysis of excluding participants with the use of anti-hypertensive drugs, hypoglycemic drugs

or lipid-lowering drugs

**Table S6** Reclassifcation and discrimination statistics for changes in cumulative TyG index

|  | **Quartiles of cumulative TyG index** | | | | **P for trend** |
| --- | --- | --- | --- | --- | --- |
|  | Q1(5.30-8.25) | Q2(8.25-8.62) | Q3(8.62-9.08) | Q4(9.08-13.03) |  |
| Case/Total | 218/14,039 | 278/14,035 | 372/14,036 | 444/14,039 |  |
| Incidence rate, per 1000 person-years | 1.57 | 2.00 | 2.69 | 3.21 |  |
| Model 1 | 1(ref) | 1.24(1.04,1.48) | 1.62(1.37,1.92) | 2.02(1.71,2.37) | <0.001 |
| Model 2 | 1(ref) | 1.09(0.91,1.30) | 1.24(1.05,1.48) | 1.28(1.07,1.53) | 0.003 |
| Model 3 | 1(ref) | 1.08(0.91,1.30) | 1.23(1.04,1.46) | 1.24(1.04,1.47) | 0.009 |

**Table S1** Association of baseline TyG index with heart failure

Model 1: adjust for age, sex.

Model 2: included variables in model 1 and further heart rate, HDL-C, LDL-C, WC, hs-CRP, eGFR, current smoker, current drinker, physical activity, hypertension, diabetes mellitus, history of myocardial infarction and history of arrhythmia.

Model 3: included variables in model 2 and further hypoglycemic drugs, anti-hypertensive drugs and lipid-lowering drugs.

**Table S2** Sensitivity analysis of excluding outcome events within the first year of follow-up (n=105)

|  | Q1 | Q2 | Q3 | Q4 | P for trend |
| --- | --- | --- | --- | --- | --- |
| Case/Total | 156/14023 | 215/14030 | 354/14007 | 482/13984 |  |
| IR | 1.09 | 1.52 | 2.57 | 3.63 |  |
|  | 1(ref) | 1.07(0.87,1.32) | 1.31(1.08,1.60) | 1.37(1.11,1.69) | 0.0006 |

Note: IR, incidence rate per 1000-person years; Model adjusted for age, sex, heart rate, HDL-C, LDL-C, WC, hs-CRP, eGFR, current smoker, current drinker, physical activity, hypertension, diabetes mellitus, history of myocardial infarction, history of arrhythmia, hypoglycemic drugs, anti-hypertensive drugs, lipid-lowering drugs and the TyG index_2010_ at baseline.

**Table S3** Sensitivity analysis of excluding participants with the history of myocardial infarction (n=100)

|  | Q1 | Q2 | Q3 | Q4 | P for trend |
| --- | --- | --- | --- | --- | --- |
| Case/Total | 153/14020 | 209/14024 | 353/14006 | 497/13999 |  |
| IR | 1.07 | 1.48 | 2.57 | 3.75 |  |
|  | 1(ref) | 1.04(0.84,1.29) | 1.32(1.08,1.61) | 1.41(1.15,1.74) | <0.0001 |

Note: IR, incidence rate per 1000-person years; Model adjusted for age, sex, heart rate, HDL-C, LDL-C, WC, hs-CRP, eGFR, current smoker, current drinker, physical activity, hypertension, diabetes mellitus, history of arrhythmia, hypoglycemic drugs, anti-hypertensive drugs, lipid-lowering drugs and the TyG index_2010_ at baseline.

**Table S4** Sensitivity analysis of competing risk model

|  | Q1 | Q2 | Q3 | Q4 | P for trend |
| --- | --- | --- | --- | --- | --- |
| Case/Total | 1640/14037 | 2318/14037 | 3141/14038 | 4001/14037 |  |
| IR | 11.72 | 16.94 | 23.70 | 31.73 |  |
|  | 1(ref) | 0.99(0.81,1.21) | 1.28(1.08,1.51) | 1.36(1.15,1.61) | <0.0001 |

Note: IR, incidence rate per 1000-person years; Model adjusted for age, sex, heart rate, HDL-C, LDL-C, WC, hs-CRP, eGFR, current smoker, current drinker, physical activity, hypertension, diabetes mellitus, history of myocardial infarction, history of arrhythmia, hypoglycemic drugs, anti-hypertensive drugs, lipid-lowering drugs and the TyG index_2010_ at baseline.

**Table S5** Sensitivity analysis of excluding participants with the use of anti-hypertensive drugs, hypoglycemic drugs

or lipid-lowering drugs.

|  | Q1 | Q2 | Q3 | Q4 | P for trend |
| --- | --- | --- | --- | --- | --- |
| Excluding participants with the use of anti-hypertensive drugs(n=9110) | | | | | |
| Case/Total | 138/12558 | 169/11893 | 264/11463 | 376/11125 |  |
| IR | 1.08 | 1.41 | 2.35 | 3.57 |  |
|  | 1(ref) | 0.99(0.80,1.26) | 1.24(0.99,1.54) | 1.38(1.10,1.73) | 0.0006 |
| Excluding participants with the use of hypoglycemic drugs(n=3466) | | | | | |
| Case/Total | 156/13782 | 192/13539 | 322/13178 | 405/12184 |  |
| IR | 1.11 | 1.41 | 2.49 | 3.51 |  |
|  | 1(ref) | 0.98(0.79,1.22) | 1.29(1.05,1.58) | 1.44(1.17,1.78) | <0.0001 |
| Excluding participants with the use of lipid-lowering drugs(n=1692) | | | | | |
| Case/Total | 155/13789 | 207/13731 | 361/13621 | 479/13316 |  |
| IR | 1.10 | 1.50 | 2.70 | 3.80 |  |
|  | 1(ref) | 1.04(0.84,1.28) | 1.37(1.12,1.67) | 1.41(1.14,1.74) | <0.0001 |

Note: IR, incidence rate per 1000-person years; Model adjusted for age, sex, heart rate, HDL-C, LDL-C, WC, hs-CRP, eGFR, current smoker, current drinker, physical activity, hypertension, diabetes mellitus, history of myocardial infarction, history of arrhythmia, hypoglycemic drugs, anti-hypertensive drugs, lipid-lowering drugs and the TyG index_2010_ at baseline.

|  | | C statistics | NRI | P | IDI | P |
| --- | --- | --- | --- | --- | --- | --- |
| Herat failure | Original model | 0.7593 | ref | ref | ref | ref |
|  | Original model+TyG_2006_ | 0.7604 | 0.1465 | <0.0001 | 0.000081 | 0.1607 |
|  | Original model+TyG_2010_ | 0.7601 | 0.0776 | 0.0067 | 0.000049 | 0.2379 |
|  | Original model+cumTyG | 0.7613 | 0.2537 | <0.0001 | 0.000077 | 0.4281 |

**Table S6** Reclassifcation and discrimination statistics for changes in cumulative TyG index

NRI net reclassification index, IDI integrated discrimination improvement, TyG index triglyceride-glucose index

The original model is the ACC/AHA model.
