# Supplementary figures and images for: Relationship between cumulative exposure to triglyceride-glucose index and heart failure: a prospective cohort study
Source: Cardiovasc Diabetol. 2023 Sep 4;22:239. doi: 10.1186/s12933-023-01967-5 (PMC10476374; doi:10.1186/s12933-023-01967-5)

**Additional file 2**

**Fig. S1** Kaplan–Meier incidence rate of heart failure by baseline TyG index


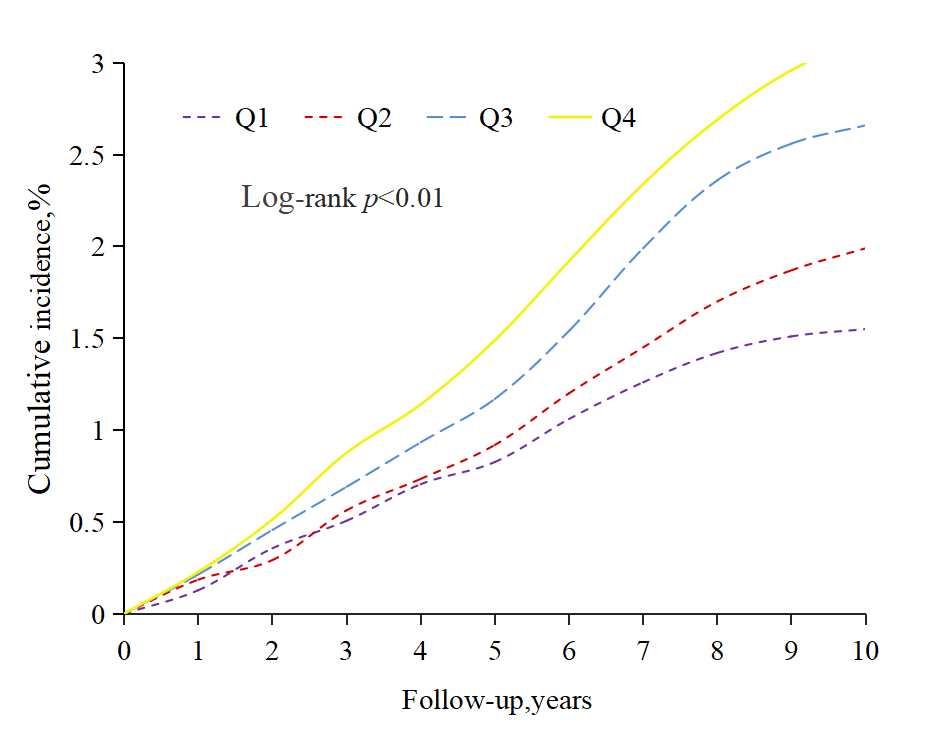

Supplement: Supplementary file 2 — Supplementary Material 2 [file 12933_2023_1967_MOESM2_ESM.docx]
